# Supplementary material for: Low-dimensional factorized neural computations underlie risk-adaptive choices
Source: Res Sq. 2026 Aug 12:rs.3.rs-10270177. Preprint. [Version 1] doi: 10.21203/rs.3.rs-10270177/v1 (PMC13640892; doi:10.21203/rs.3.rs-10270177/v1)
Supplement: Supplementary file 1 [file NIHPPRS10270177V1-supplement-1.pdf]

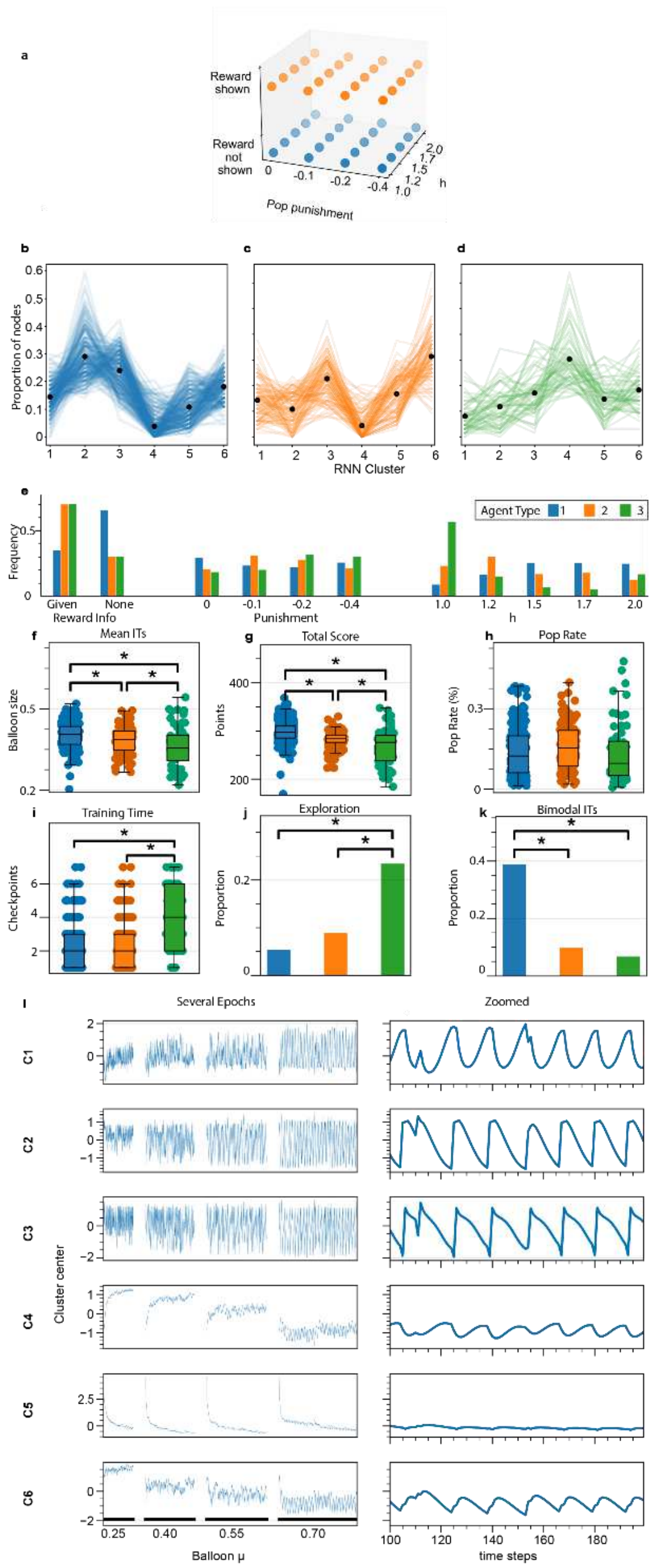

Supplementary Figure 1. Representative activations for deep RL agent node clusters. a, training parameters for the agents. **b-d,** Proportion of nodes from each of the three agent types. Type 1 (**b**) are very similar to Type 2 (**c**), with the exception of relative proportion of node type 2. Type 3 agents are pictured in (**d**). **b,d**, similar to Fig 1. **e,** Frequency of agent types that developed based on different task parameters. Type 1 = Bimodal, Type 3 = Explorer. **f**, Mean inflation time for each of the agents, paired t-tests. **g**, Total score for each agent type, paired t-tests. **h**, Pop rate for each agent type, **i**, Number of training checkpoints until performance level achieved, paired t-tests. **j**, Exploration metric for each type of agent type, paired t-tests **k,**

Proportion of agents with bimodal inflation times, paired t-tests. **I,** Examples of six clusters of node activities. The left column shows node activity across several epochs. The right column shows node activity across approximately 100 time steps. *, **, *** indicate statistical significance (p<0.05, p<0.01, p<0.001)

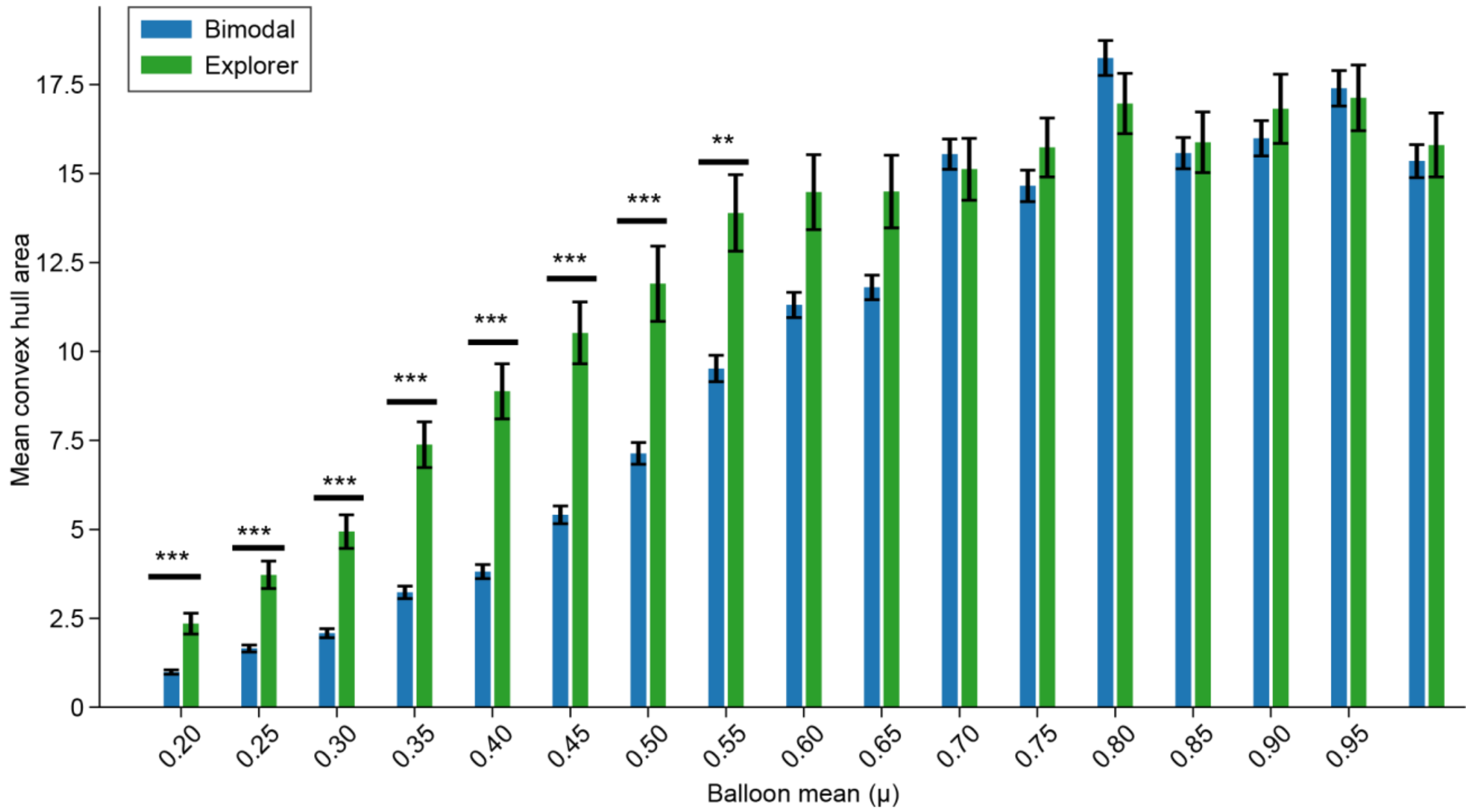

Supplemental Figure 2. Surface area of neural trajectory arcs in deep RL agents.
Measures of the area of the convex hull of the trajectories in PCA space of Bimodal and Explorer agents. Error bars are SEM. *, **, *** indicate statistical significance ($p<0.05$, $p<0.01$, $p<0.001$).

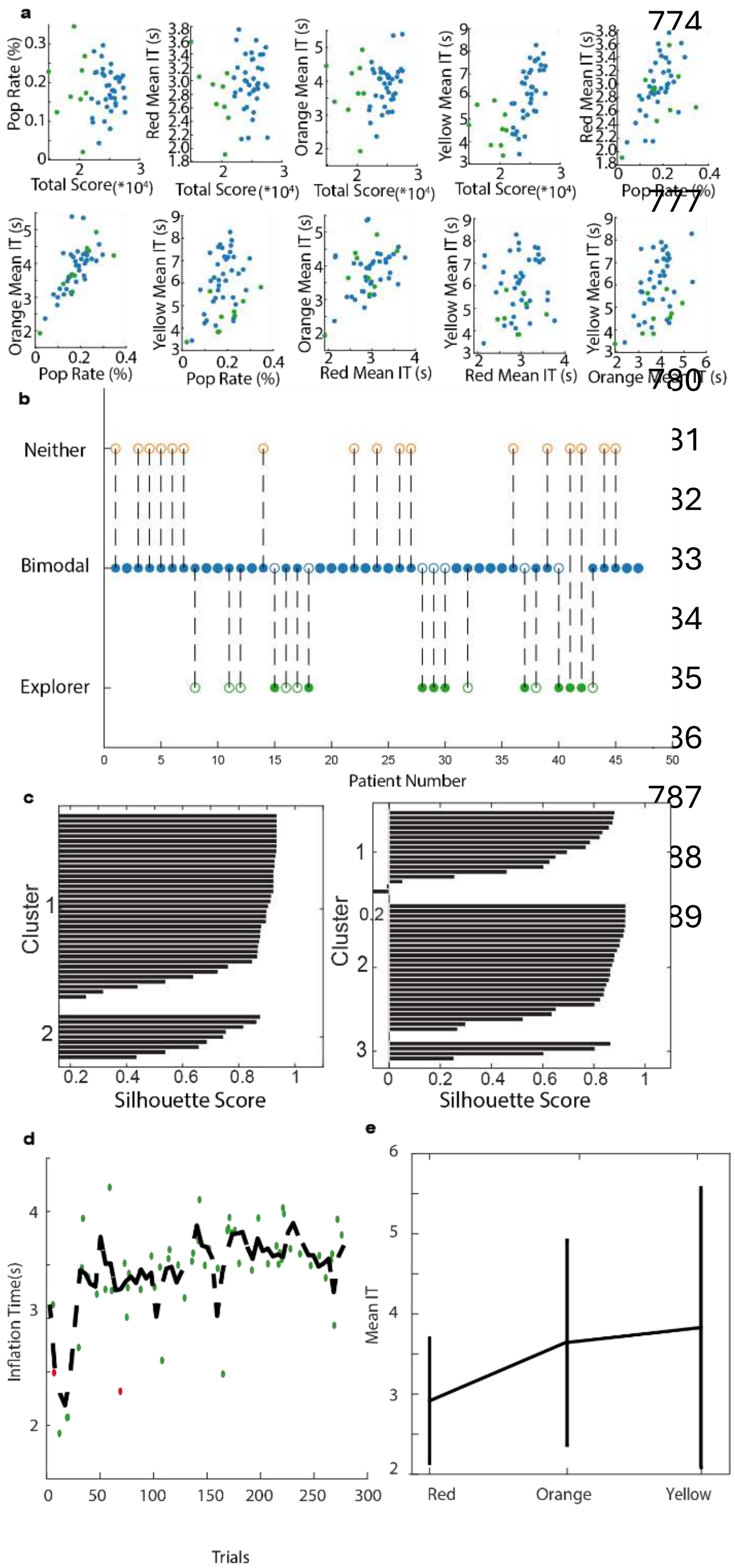

Supplementary Figure 3. Clustering human risky choice behavior. a, Behavioral measures on which k-means clustering was done. blue corresponds to Bimodal, green corresponds to Explorer **b,** Open circles indicate the original assignment of the patient based solely on behavioral metrics. Closed circles indicate the final categorization of the patient based on k-means clustering. **c,** Silhouette scores of k-means clustering. k = 2 led to better silhouette scores than k = 3, so k = 2 was used and the 'Neither' categorization was dropped. **d**, an example Bimodal patient where yellow balloon sizes were learned after only a few trials. **e**, Mean inflation times for red, orange and yellow balloons respectively for an example Explorer human.

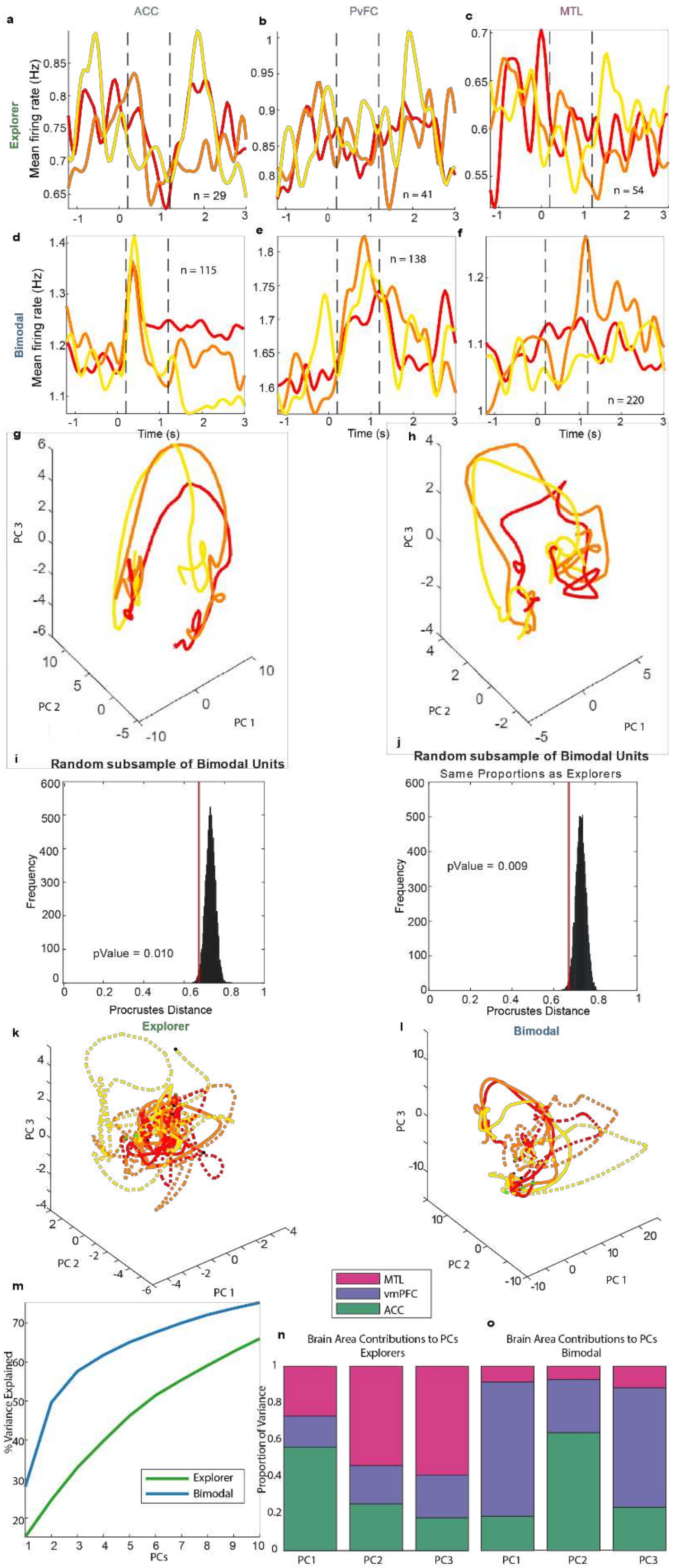
ACC
PvFC
MTL
Explorer
Mean firing rate (Hz)
n = 29
n = 41
n = 54
Bimodal
n = 115
n = 138
n = 220
Time (s)
PC 1
PC 2
PC 3
Random subsample of Bimodal Units
Same Proportions as Explorers
Frequency
pValue = 0.010
pValue = 0.009
Procrustes Distance
Explorer
Bimodal
% Variance Explained
PCs
MTL
vmPFC
ACC
Brain Area Contributions to PCs
Explorers
Brain Area Contributions to PCs
Bimodal
Proportion of Variance
PC1
PC2
PC3

Supplemental Figure 4. Features of human neuron dynamical computation. a-f, Trial-averaged firing rates by behavioral class and brain region. Dashed lines mark the 200 ms -1200 ms *a priori* window. **g-h,** PCA trajectories of neural trajectories from subsampled Bimodal pseudopopulation. The relative proportions of units sampled from each brain area were held consistent with the Explorer pseudopopulation. **i,j,** Permutation tests for Procrustes Distance metric comparing the geometries of subsampled neurons from the Bimodal and Explorer groups. **k,** the Bimodal population of units was subsampled without replacement such that the subpopulation had the same number of units as the Explorer group. **l,** the Bimodal population of units was subsampled such that the subpopulation had the same number of units as the Explorer group and that the proportion of units from each brain area matched (i.e. the subpopulation of Bimodal units contained exactly 29 units). **k-l,** 3D cue-aligned PCA trajectories for the pseudopopulations of Explorer (**k**) and Bimodal (**l**) with active trials represented as solid lines and passive trials represented as dashed lines. **m**, Variance explained for PCA on cue-aligned firing rates for Bimodal and Explorer humans. **n-o**, Proportion of variance explained by each brain area grouped by PC for Explorers (**n**) and Bimodal (**o**) pseudoensembles (sum of squared loadings).

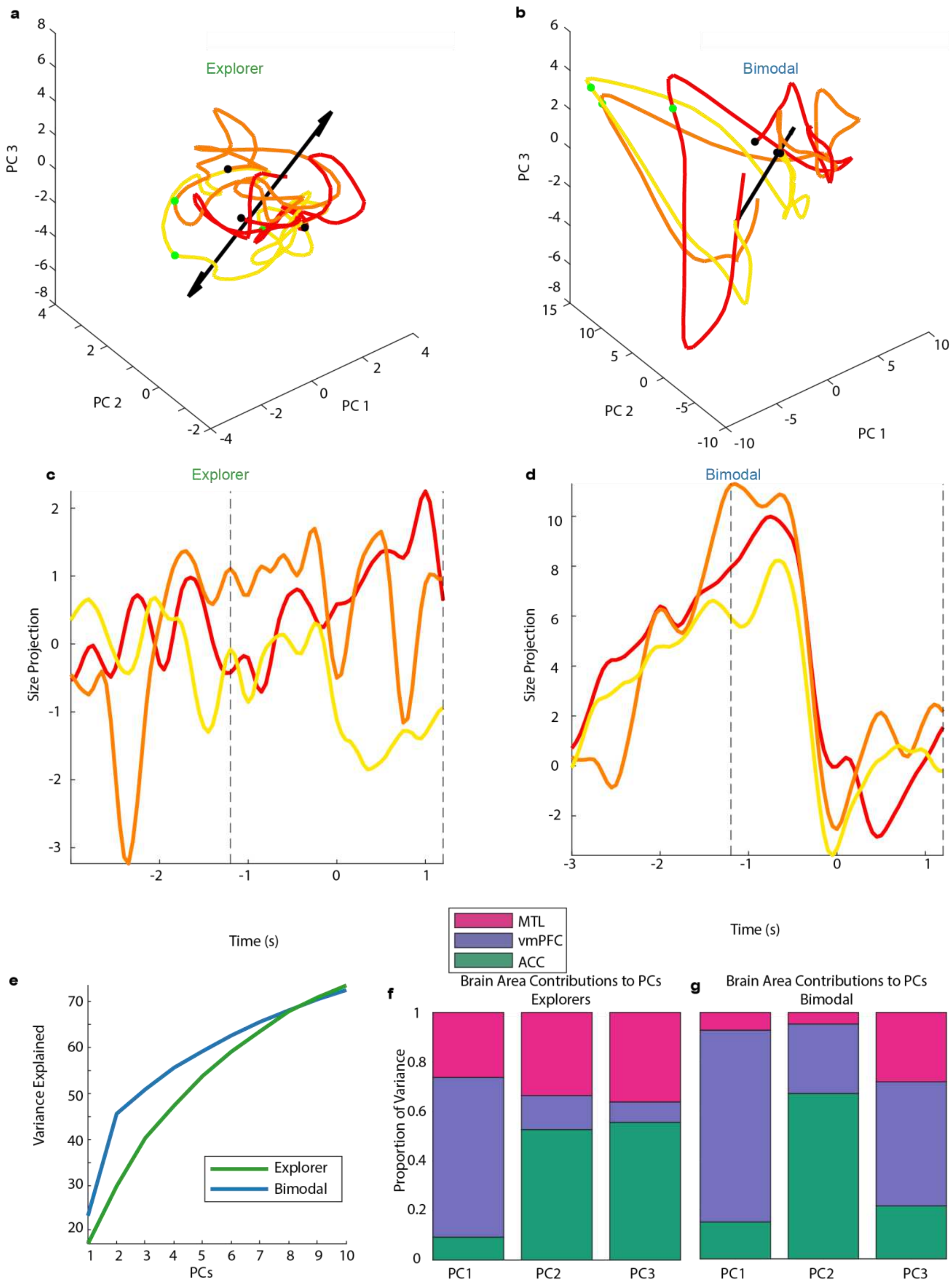
a
Explorer
PC 3
PC 2
PC 1
b
Bimodal
PC 3
PC 2
PC 1
c
Explorer
Size Projection
Time (s)
d
Bimodal
Size Projection
Time (s)
MTL
vmPFC
ACC
e
Variance Explained
PCs
Explorer
Bimodal
f
Brain Area Contributions to PCs
Explorers
Proportion of Variance
PC1
PC2
PC3
g
Brain Area Contributions to PCs
Bimodal
PC1
PC2
PC3

Supplemental Figure 5. Outcome-aligned neural trajectories. a,b Outcome aligned neural trajectories in 3D PCA space for humans using Explorer (**a**) and Bimodal (**b**) strategies. Black arrows are decoded size axes, black dots are beginning of recording window and green dot is when outcome occurs. **c-d,** Trajectories projected onto the Size Axis. Dotted lines bracket (± 1200 ms)the moment that outcome occurs. **e,** Variance explained for 10 PCs. **f-g,** Proportion of variance explained by each brain area grouped by PC for Explorers (**f**) and Bimodal (**g**) pseudoensembles (sum of squared loadings).
